# Associations of Culprit Vessel Size and Plaque Characteristics in Patients with ST-Segment Elevation Myocardial Infarction

**DOI:** 10.31083/j.rcm2407186

**Published:** 2023-06-29

**Authors:** Jiannan Li, Runzhen Chen, Jinying Zhou, Ying Wang, Xiaoxiao Zhao, Chen Liu, Peng Zhou, Yi Chen, Li Song, Shaodi Yan, Hongbing Yan, Hanjun Zhao

**Affiliations:** ^1^Department of Cardiology, Fuwai Hospital, National Center for Cardiovascular Diseases, Peking Union Medical College and Chinese Academy of Medical Sciences, 100005 Beijing, China; ^2^Fuwai Hospital, Chinese Academy of Medical Sciences, 518057 Shenzhen, Guangdong, China; ^3^Coronary Heart Disease Center, Fuwai Hospital, Chinese Academy of Medical Sciences, 100021 Beijing, China

**Keywords:** small vessel disease, optical coherence tomography, plaque rupture, plaque erosion

## Abstract

**Background:**

Small vessel disease (SVD) widely exists in patients with acute coronary syndrome. However, the plaque characteristic of SVD has not been investigated.

**Methods:**

Optical coherence tomography (OCT) of culprit lesion was examined in 576 patients with ST-segment elevation myocardial infarction (STEMI) and finally 404 patients with qualified images were analysed of plaque phenotypes and microstructure. The cohort was divided into three groups according to vessel diameters of culprit lesion which were measured by OCT. Major adverse cardiac events (MACEs) were recorded of each patient and compared among patients with different vessel diameters and plaque phenotypes.

**Results:**

Gender, age and body mass index (BMI) were significantly different among patients with different diameters of culprit vessels (98.4% vs. 85.7% vs.71.4%, *p* < 0.001; 40.0 ± 7.0 vs. 54.9 ± 6.6 vs. 68.9 ± 5.8, *p* < 0.001; 28.4 ± 4.0 vs. 25.8 ± 2.9 vs. 25.2 ± 3.0, *p* < 0.001, respectively). Moreover, patients with diameters of culprit lesion >3 mm presented with more incidence of plaque rupture and macrophage (57.7% vs. 42.1% vs. 46.2%, *p* = 0.015, 55.1% vs. 41.0% vs. 36.9%, *p* = 0.010). Total MACE did not differ among groups of different vessel diameters and plaque phenotypes.

**Conclusions:**

Vessel size of culprit lesion is significantly associated with plaque phenotype in patients with STEMI. However, patients with different diameters and plaque phenotypes showed no significant difference of clinical outcomes.

**Clinical Trial Registration:**

NCT03593928.

## 1. Introduction

Small vessel disease (SVD) has emerged as an intriguing issue of atherosclerotic coronary artery disease nowadays. SVD was reported to account for 30% to 67% of patients undergoing percutaneous coronary intervention (PCI) [1, 2]. Moreover, SVD also existed in patients with acute coronary syndrome (ACS), or even ST-segment elevation myocardial infarction (STEMI) [3, 4, 5]. Compared with large vessel disease (LVD), SVD had poorer prognosis [6] and more easily appeared in female patients with diabetes and chronic kidney disease [6, 7]. Although drug eluting stent (DES), drug coated balloon (DCB) or bioresorbable scaffolds (BRS) were proven to be effective for treating SVD [8, 9], the most challenge issue of SVD is equivocal definition and optimal treatment which depended on more refined classification.

Although previous observation studies illustrated the clinical features of SVD, whether the intravascular structure in lesions of small vessels differs from large vessels remained unknown. Moreover, the relationship between plaque morphology and vessel size was not investigated yet. Optical coherence tomography (OCT) is one of the powerful intracoronary image devices which not only identified plaque characteristics but also determined lumen size of culprit lesion more accurately than intravenous ultrasound (IVUS) or quantitative coronary analysis (QCA), especially in small coronary vessels [10, 11]. Herein, we measured diameters of culprit lesion determined by OCT and investigated its association with plaque characteristics.

## 2. Methods

### 2.1 Study Population

**Fig. 1.**
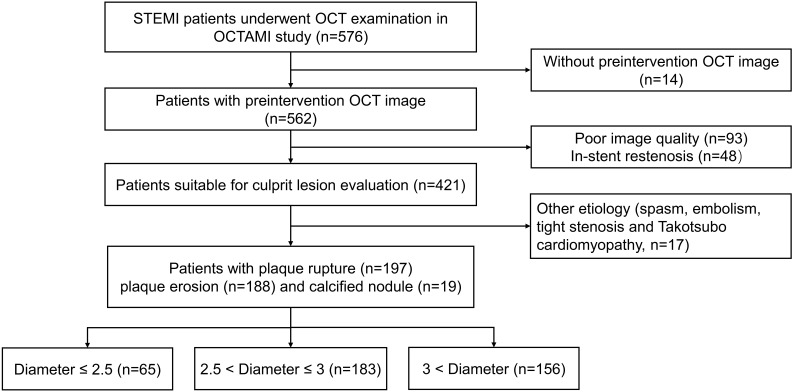
**Study flow chart**. OCT, optical coherence tomography; STEMI, ST-segment elevation myocardial infarction; OCTAMI, Optical Coherence Tomography Examination in Acute Myocardial Infarction.

From March 2017 to January 2020, 576 patients with STEMI who underwent OCT imaging of culprit lesions in Fuwai Hospital were consecutively recruited (Fuwai Hospital Optical Coherence Tomography Examination in Acute Myocardial Infarction (OCTAMI) Registry, clinical trials.gov: NCT03593928). After excluding patients without preintervention OCT images (n = 14), patients with poor OCT image quality (n = 93), patients with in-stent restenosis (n = 48), patients with other etiology of ACS (n = 17), the remaining 404 patients with plaque rupture (n = 197), plaque erosion (n = 188) and calcified nodules (n = 19) were ultimately included for analysis. The study flow chart is displayed in Fig. 1. This study was performed in accordance with the Declaration of Helsinki and was approved by the Ethics Committee of Fuwai Hospital. All patients provided written informed consent.

### 2.2 OCT Image Acquisition and Analysis

Patients were administered 300 mg aspirin, 180 mg ticagrelor, or 600 mg clopidogrel, and 100 IU/kg heparin before the interventional procedure. Percutaneous coronary intervention was performed via radial or femoral access. Thrombus aspiration was used to reduce the thrombus burden and restore the antegrade coronary flow. OCT images of the culprit lesions were acquired with the frequency domain ILUMIEN OPTIS OCT system and a dragon fly catheter (St. Jude Medical, Westford, MA) after the antegrade blood flow was restored, according to the intracoronary imaging technique previously described. Intracoronary injection of nitroglycerin is generally performed before OCT imaging in most of cases except hypotension. Reference vessel diameter (RVD) of culprit lesions was evaluated by OCT before intervention. RVD was determined where at least 180° of external elastic lamina (EEL) could be visualized within 5 mm from target lesion [12]. If EEL is invisible, RVD was estimated on basis of comparatively regular vessel segment besides culprit lesion or according to diameters of stent.

**Fig. 2.**
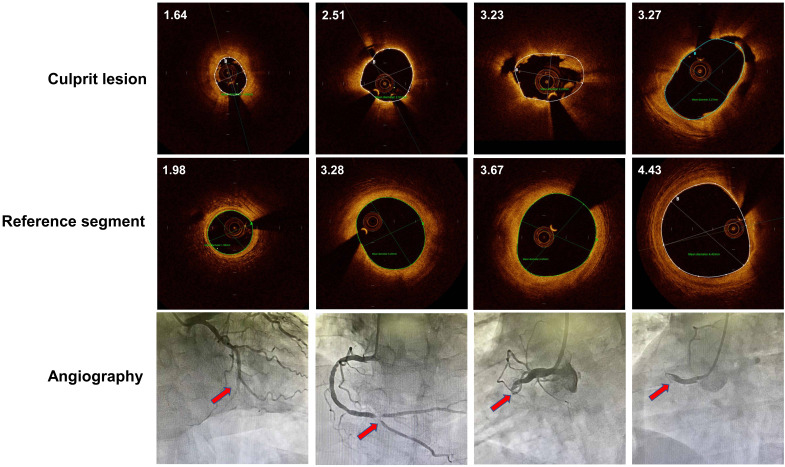
**OCT image of culprit lesion and reference segment with small to large diameters**. Every column indicated representative OCT and angiography images indicating luminal size from left to right. Mean diameters of coronary lumen were labelled on the left upper corner of OCT images. Red arrow indicated culprit lesion site. OCT, optical coherence tomography

All OCT images were anonymously analysed on a St Jude OCT Offline Review Workstation by 3 independent investigators blinded to the other data. According to the previously established criteria [13], plaque rupture (PR) was identified by a disrupted fibrous cap with clear cavity formation. Thin-cap fibroatheroma (TCFA) was defined as LRP with the thinnest part of the fibrous cap being <65 µm. The fibrous cap thickness was measured in triplicate at the thinnest part of the fibrous cap of the culprit plaque, and the average value was calculated. The length of the culprit lesion was measured as the span of the entire culprit plaque in the longitudinal view. Calcification within plaques was identified by the presence of well-delineated, low-backscattering heterogeneous regions. Microchannels were defined as tubule luminal structures without a connection to the vessel lumen that did not produce a signal that was recognized in more than three consecutive cross-sectional OCT images. Cholesterol crystals were defined as linear, highly backscattering structures within the plaque. Macrophage infiltration was defined as signal-rich, distinct or confluent punctate regions above the intensity of background speckle noise with backwards shadowing. The minimal lumen area (MLA) was evaluated along the length of the target lesion. The typical OCT image of culprit lesion with small to large diameters were displayed in Fig. 2.

### 2.3 MACEs and Follow-Up

Major adverse cardiac events (MACEs) were defined as composite of all-cause death, recurrence of myocardial infarction, heart failure, stroke and unplanned revascularization. Follow-up was performed by well-trained physicians who were blinded to the routine clinical data at 1, 6, and 12 months after discharge via outpatient visits or phone interviews and then annually after 1-year follow-up.

### 2.4 Statistical Analysis

Continuous data were presented as the means ± standard deviations (SDs) or medians (interquartile ranges [IQRs]). Comparisons between two groups were performed using Student’s *t* test or the Manne Whitney U test. Categorical variables were presented as numbers and percentages. Comparisons of the frequency between two groups were performed using Pearson’s chi-square test or Fisher’s exact test. Logistic regression analysis was performed to determine the odds ratio (OR) and 95% confidence interval (CI) for healed plaque stratified according to stratification of diameters. Adjustments were made for traditional risk factors (sex, age, body mass index, current smoking, hypertension, diabetes, low-density lipoprotein-cholesterol, triglycerides, and high sensitivity C-reactive protein). A two-tailed *p* value < 0.05 was considered indicative of statistical significance. The statistical analyses were performed using SPSS software, version 25 (IBM, Armonk, NY, USA).

## 3. Results

### 3.1 Clinical Characteristics

According to the diameter of vessel in culprit lesion, patients were divided into three groups of diameter (D) ≤2.5 mm (n = 65), 2.5 mm < D ≤ 3 mm (n = 183), 3 mm< D (n = 156). In patients with STEMI, the prevalence of SVD account for 16.1% for standard of smaller than 2.5 mm and 61.4% for standard of smaller than 3 mm. Baseline characteristics of patients in each group are summarized in Table 1. The proportion of men (98.4% in diameter ≤2.5 mm to 71.4% in diameter >3 mm) significantly decreased and age (40.0 ± 7.0 in diameter ≤2.5 mm to 68.9 ± 5.8 in diameter >3 mm) significantly increased with advanced diameters of culprit lesion. BMI (28.4 ± 4.0 in diameter ≤2.5 mm to 25.2 ± 3.0 in diameter >3 mm), total cholesterol (4.8 [4.2–5.4] in diameter ≤2.5 mm to 4.2 [3.6–4.9] in diameter >3 mm), triglycerides (2.0 [1.3–3.0] in diameter ≤2.5 mm to 1.2 [0.8–1.7] in diameter >3 mm) and low density lipoprotein cholesterol (LDL-C) (3.0 [2.3–3.6] in diameter ≤2.5 mm to 2.6 [2.0–3.1] in diameter >3 mm) were lower, and previous PCI (4.6% in diameter ≤2.5 mm to 14.1% in diameter >3 mm), high density lipoprotein cholesterol (HDL-C) (1.0 [0.9–1.2] in diameter ≤2.5 mm to 1.1 [1.0–1.3] in diameter >3 mm) were higher in larger diameters group. Moreover, small vessel lesion usually occurred in left circumfex artery (LCX) (40.0% in diameter ≤2.5 mm to 2.6% in diameter >3 mm) while infrequently occurred in right coronary artery (RCA) (16.9% in diameter ≤2.5 mm to 61.5% in diameter >3 mm).

**Table 1.** **Baseline clinical characteristics**.

Variables				Group 1	Group 2	Group 3	*p* value			
				D ≤2.5 (n = 65)	2.5< D ≤3.0 (n = 183)	3.0< D (n = 156)	overall	1 vs. 2	1 vs. 3	2 vs.3
Patient characteristics										
	Age (mean ± SD)			40.0 ± 7.0	54.9 ± 6.6	68.9 ± 5.8	<0.001	<0.001	<0.001	<0.001
	Male, n (%)			62 (98.4)	150 (85.7)	105 (71.4)	<0.001	0.006	<0.001	0.002
	BMI (mean ± SD)			28.4 ± 4.0	25.8 ± 2.9	25.2 ± 3.0	<0.001	<0.001	<0.001	0.126
Past history										
	Smoking, n (%)			52 (80.0)	137 (75.3)	103 (66.0)	0.055	0.440	0.039	0.062
	Hypertension, n (%)			37 (56.9)	99 (54.1)	98 (62.8)	0.264	0.694	0.413	0.105
	Dyslipidemia, n (%)			58 (89.2)	164 (89.6)	138 (88.5)	0.943	0.930	0.869	0.734
	Diabetes mellitus, n (%)			17 (26.2)	56 (30.6)	49 (31.4)	0.731	0.499	0.437	0.872
	Stroke, n (%)			0 (0)	17 (9.3)	20 (12.8)	0.011	0.011	0.002	0.307
	CKD, n (%)			2 (3.1)	0 (0)	7 (4.5)	0.018	0.017	0.629	0.004
	Myocardial infarction, n (%)			2 (3.1)	11 (6.0)	18 (11.5)	0.051	0.362	0.046	0.070
	PCI, n (%)			3 (4.6)	11 (6.0)	22 (14.1)	0.014	0.675	0.042	0.012
Laboratory data										
	Total cholesterol (mmol/L)			4.8 (4.2–5.4)	4.4 (3.8–5.1)	4.2 (3.6–4.9)	<0.001	0.003	<0.001	0.116
	LDL cholesterol (mmol/L)			3.0 (2.3–3.6)	2.8 (2.4–3.3)	2.6 (2.0–3.1)	0.006	0.208	0.005	0.017
	HDL cholesterol (mmol/L)			1.0 (0.9–1.2)	1.1 (0.9–1.2)	1.1 (1.0–1.3)	0.010	0.086	0.003	0.071
	TG (mmol/L)			2.0 (1.3–3.0)	1.5 (1.0–2.0)	1.2 (0.8–1.7)	<0.001	0.002	<0.001	0.001
	Serum creatinine (mmol/L)			83.8 (76.3–93.1)	78.6 (68.3–91.7)	83.3 (70.4–94.2)	0.031	0.020	0.668	0.040
	WBC (${\mathrm{10}}^{9}$/L)			10.7 ± 3.6	10.0 ± 2.9	9.4 ± 2.7	0.007	0.126	0.003	0.039
	hs-CRP (mg/dL)			8.5 (4.0–10.9)	5.7 (2.3–10.9)	5.8 (2.5–10.7)	0.375	0.185	0.222	0.784
	HbA1c (%)			6.6 ± 1.6	6.6 ± 1.6	6.6 ± 1.4	0.906	0.846	0.890	0.658
	cTNI (ng/mL)			1.1 (0.2–5.3)	1.3 (0.1–4.9)	0.8 (0.1–5.9)	0.436	0.761	0.275	0.287
	NT-proBNP (pg/mL)			109.1 (32.3–353.5)	179.9 (45.4–507.6)	240.0 (83.2–850.0)	0.002	0.064	0.001	0.021
	LVEF (%)			55.5 ± 4.5	54.8 ± 6.2	54.5 ± 6.7	0.515	0.421	0.250	0.622
Angiography data										
	Culprit vessel						<0.001	<0.001	<0.001	<0.001
		LAD, n (%)		28 (43.1)	107 (58.5)	56 (35.9)				
		LCX, n (%)		26 (40.0)	15 (8.2)	4 (2.6)				
		RCA, n (%)		11 (16.9)	60 (32.8)	96 (61.5)				
	TIMI flow						0.406	0.692	0.899	0.122
		0		42 (64.6)	105 (57.4)	106 (67.9)				
		1		2 (3.1)	10 (5.5)	5 (3.2)				
		2		7 (10.8)	26 (14.2)	12 (7.7)				
			3	14 (21.5)	42 (23.0)	33 (21.2)				
	Stent, n (%)			55 (84.6)	177 (96.7)	152 (97.4)	<0.001	0.001	<0.001	0.698
	IABP, n (%)			0 (0)	4 (2.2)	3 (1.9)	0.497	0.229	0.260	0.865
Medical therapy										
	Aspirin			62 (95.4)	177 (96.7)	154 (98.7)	0.314	0.621	0.129	0.227
	Clopidogrel			29 (44.6)	87 (47.5)	80 (51.3)	0.624	0.685	0.366	0.492
	Ticagrelor			36 (55.4)	95 (51.9)	77 (49.4)	0.708	0.630	0.414	0.639
	ACEI/ARB			51 (78.5)	137 (74.9)	112 (71.8)	0.568	0.561	0.305	0.524
	β-blocker			61 (93.8)	163 (89.1)	132 (84.6)	0.134	0.263	0.060	0.224
	Statin			64 (98.5)	176 (96.2)	153 (98.1)	0.458	0.370	0.845	0.302
	Anticoagulant			4 (6.2)	2 (1.1)	2 (1.3)	0.031	0.023	0.042	0.872
	PPI			29 (44.6)	85 (46.4)	79 (50.6)	0.637	0.799	0.414	0.441

Continuous data are presented as median (interquartile range). Categorical data are presented as number (%). D, diameter; BMI, body mass index; PCI, percutaneous coronary intervention; CKD, chronic kidney disease; WBC, white blood cell; HDL, high density lipoprotein; LDL, low density lipoprotein; TG, triglyceride; hs-CRP, high sensitive C-reactive protein; HbA1c, Hemoglobin A1c; cTNI, cardiac troponin I; LVEF, left ventricle ejection fraction; LAD, left anterior descending artery; LCX, left circumfex artery; RCA, right coronary artery; TIMI, thrombolysis in myocardial infarction; IABP, Intra-aortic balloon pump; ACEI, angiotensin-converting enzyme inhibitor; ARB, angiotensin receptor blocker; PPI, proton pump inhibitors; NT-proBNP, N-terminal pro-B-type natriuretic peptide.

### 3.2 OCT Findings

The proportion of plaque rupture is significantly higher in group of diameters larger than 3 mm than that between 2.5 mm and 3 mm (57.1% vs. 42.1%, *p* = 0.004) whereas the presence of plaque erosion is lower in group 3 than group 2 (53.6% vs. 36.5%, *p* = 0.002). The prevalence of calcified nodule is similar among three groups.

In microstructure of plaque, MLA and presence of macrophage was significant different among three groups (1.5 [1.2–2.0] vs. 1.7 [1.3–2.1] vs. 2.0 [1.5–2.4], *p* < 0.001; 36.9% vs. 41.0% vs. 55.1%, *p* = 0.010, respectively). The incidence of TCFA is significantly higher in group 3 than group 2 (34.6% vs. 24.0%, *p* = 0.032) (Table 2).

**Table 2.** **OCT findings**.

OCT features		Group 1	Group 2	Group 3	*p* value			
		D ≤2.5 (n = 65)	2.5< D ≤3.0 (n = 183)	3.0< D (n = 156)	Overall	1 vs. 2	1 vs. 3	2 vs.3
Plaque phenotypes								
	Plaque rupture	30 (46.2)	77 (42.1)	90 (57.7)	0.015	0.569	0.117	0.004
	Plaque erosion	33 (50.8)	98 (53.6)	57 (36.5)	0.006	0.699	0.050	0.002
	Calcified nodule	2 (3.1)	8 (4.4)	9 (5.8)	0.662	0.649	0.402	0.557
Microstructure								
	Lipid plaque, n (%)	30 (46.2)	90 (49.2)	85 (54.5)	0.449	0.675	0.259	0.330
	Fibrous plaque, n (%)	30 (46.2)	70 (38.3)	53 (34.0)	0.233	0.265	0.088	0.414
	Calcification, n (%)	28 (43.1)	91 (49.7)	83 (53.2)	0.388	0.357	0.170	0.523
	Microchannel, n (%)	13 (20.0)	36 (19.7)	34 (21.8)	0.884	0.955	0.766	0.630
	Cholesterol crystal, n (%)	6 (9.2)	23 (12.6)	24 (15.4)	0.446	0.472	0.224	0.455
	Macrophage, n (%)	24 (36.9)	75 (41.0)	86 (55.1)	0.010	0.566	0.014	0.009
	Thrombus, n (%)	62 (95.4)	179 (97.8)	154 (98.7)	0.310	0.310	0.129	0.529
	TCFA, n (%)	15 (23.1)	44 (24.0)	54 (34.6)	0.061	0.875	0.092	0.032
	FCT, µm	100 (70–130)	100 (60–120)	90 (60–120)	0.177	0.421	0.090	0.182
	MLA, ${\mathrm{mm}}^{2}$	1.5 (1.2–2.0)	1.7 (1.3–2.1)	2.0 (1.5–2.4)	<0.001	0.077	<0.001	<0.001

Continuous data are presented as median (interquartile range). Categorical data are presented as number (%). D, diameter; TCFA, thin-cap fibroatheroma; FCT, fibrous cap thickness; MLA, minimal lumen area; OCT, optical coherence tomography.

### 3.3 Follow-Up Analysis

**Fig. 3.**
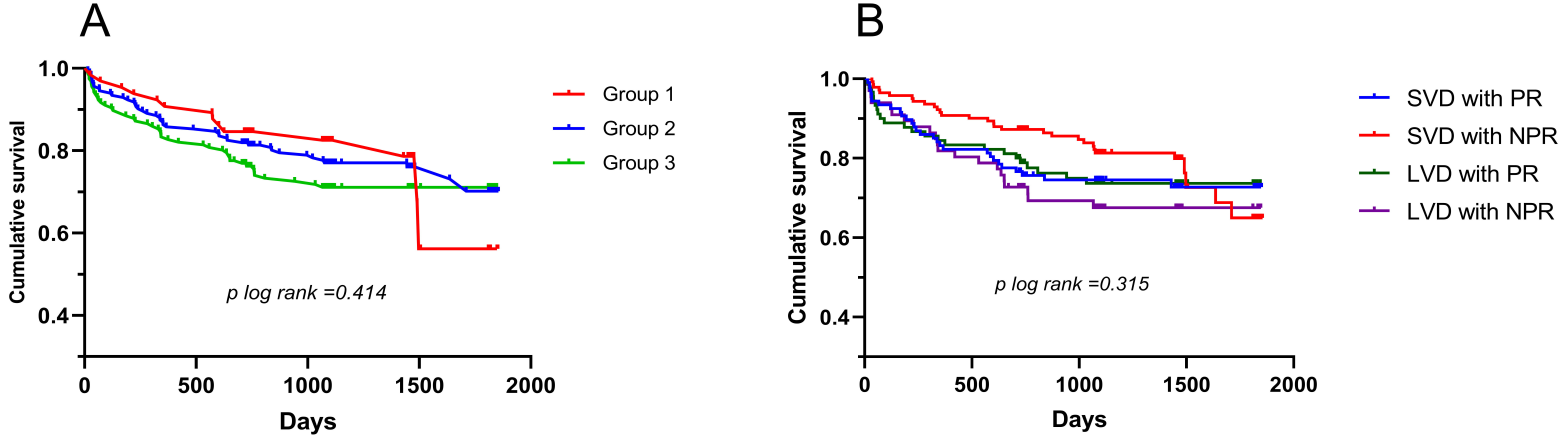
**Kaplan-Meier curve for patients with different diameters of culprit lesion (A) and groups which divided by vessel size and plaque phenotypes (B)**. SVD, small vessel disease; LVD, large vessel disease; PR, plaque rupture; NPR, nonplaque rupture.

The median time to follow-up was 3 years (interquartile range: 2 to 4 years). A KM curve was drawn according to the luminal diameters of the culprit lesion. No significant difference in MACEs was observed among three groups. In addition, the cohort was divided into 4 groups according to vessel size and plaque phenotype: SVD (diameter ≤3 mm) with plaque rupture (PR), SVD with non-plaque rupture (NPR), LVD (diameter >3 mm) with PR and LVD with NPR. No significant difference in MACEs was observed among four groups (Fig. 3).

### 3.4 Multivariate Logistic Regression Analysis

**Fig. 4.**
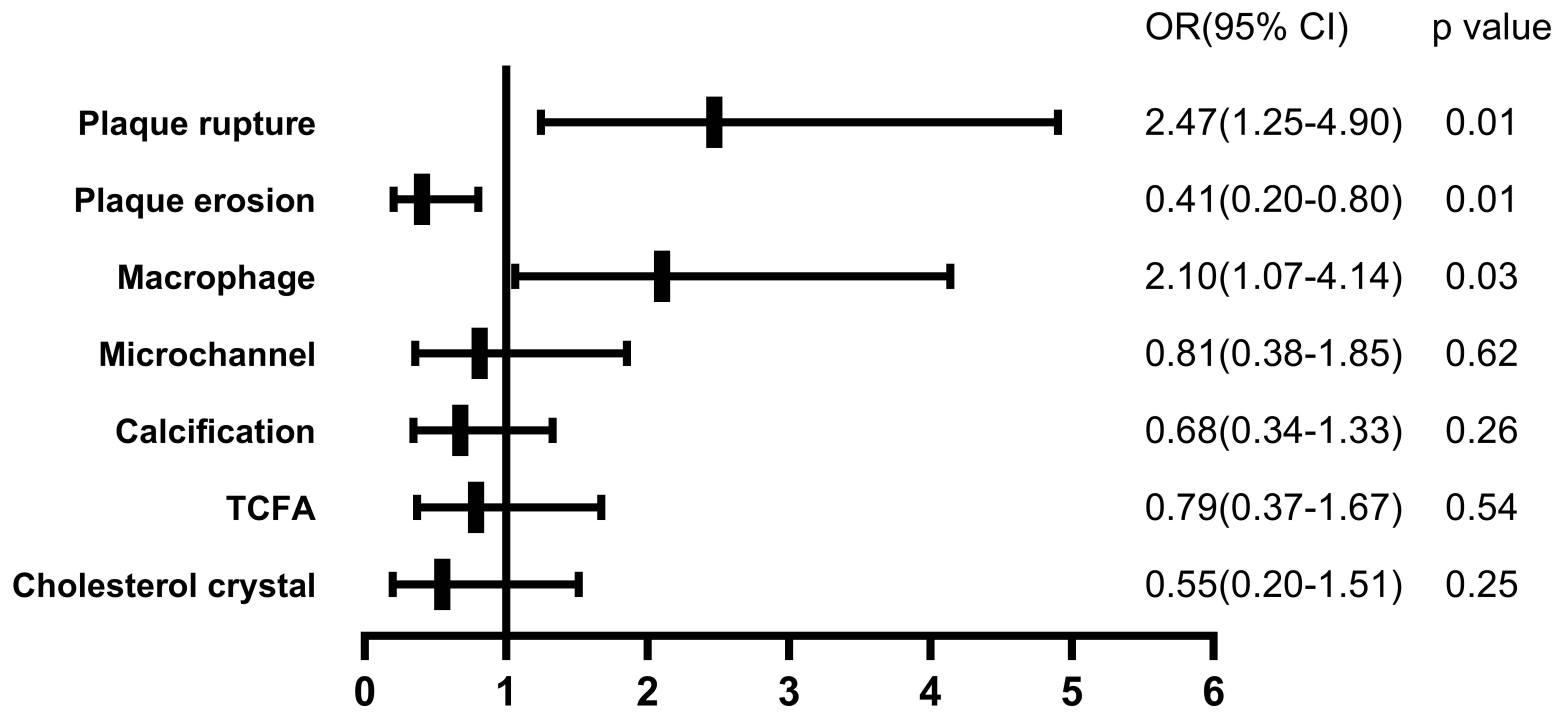
**The impact of diameters of culprit lesion on plaque features, adjusted for patient characteristics**. After adjusting patient characteristics (sex, age, body mass index, current smoking, hypertension, diabetes, low-density lipoprotein-cholesterol, triglycerides, and high sensitivity C-reactive protein), patients with diameters >3 mm were associated with the presence of plaque rupture and macrophage. OR, odds ratio; TCFA, thin-cap fibroatheroma.

Due to the similar clinical features and OCT findings of group 1 and 2, the cohort was divided into two groups with diameters >3 mm and ≤3 mm. After adjusting risk factors, diameters of culprit lesion were significantly related to the presence of plaque rupture (OR, 2.471; 95% CI, 1.246–4.900; *p* = 0.01), plaque erosion (OR, 0.405; 95% CI, 0.204–0.803; *p* = 0.01) and macrophage (OR, 2.101; 95% CI, 1.066–4.141; *p* = 0.032) (Fig. 4).

## 4. Discussion

This single centre study summarized clinical and intracoronary features of different culprit lesion size in patients with STEMI. The main results of the current study showed that patients with diameters >3 mm of culprit lesion tended to be female, older and thinner. Moreover, patients with larger lumen size presented with more incidence of plaque rupture and macrophage. In addition, patients with culprit diameters ≤2.5 mm exhibited similar clinical manifestation and OCT features with patients with 2.5 mm ≤ diameters < 3 mm. The 3-year clinical outcome showed no significant difference among 3 groups.

In the past few decades, SVD has emerged as an intriguing issue of atherosclerotic coronary artery disease. Although abundant therapeutic methods were discovered, the optimal treatment option remained conflicting in various clinical trials. In patients with de novo lesion <3 mm, DCB and second-generation DES showed similar clinical outcomes at 12 months [14]. In contrast, in a recent retrospective study, patients treated with DCB in vessels ≤2.5 mm had significantly higher incidence of restenosis than DES [15]. The prime cause of this situation is that no uniform standard of SVD was determined previously and the diameters of SVD spanned from smaller than 2.25 mm to 3.0 mm in various studies [14, 16, 17]. Moreover, the measurement approach of vessel diameters was discordant in different studies, including angiographic or intracoronary quantification and even visual estimation [18]. On the other hand, SVD also presented with distinct plaque morphology and clinical manifestation, which should be taken into consideration in decision process. For example, SVD patients with diabetes represent much more high-risk than those without diabetes [19]. Furthermore, DCBs were more beneficial for patients with diabetes than DESs [20]. However, *in vivo* data about association of SVD and plaque morphology are lacking.

Plaque rupture, plaque erosion and calcified nodule are three main pathological phenotypes of ACS which presented different clinical outcome [21]. Although previous study reported that direct stenting had superior clinical outcome than conventional stenting in patients of STEMI with SVD [4], a recent OCT study demonstrated that ACS patients with plaque erosion benefited from medical therapy without stenting, including SVD [22]. Also, some case reports showed that patients with AMI and plaque erosion accepted successful treatment of thrombus aspiration and balloon without stent under OCT guidance [23]. However, there is still no evidence of safety and efficacy of no stent strategy for patients with plaque rupture. The research of drug-coated balloons treating vulnerable plaques is currently ongoing [24]. For calcified lesion, smaller balloons at higher pressures without coronary injuries was needed before stent implantation [25]. In this study, we found significantly different distribution of plaque phenotypes in patients with small to large diameters of culprit lesion. SVD patients with STEMI were easier to present plaque erosion, which may provide important clues for precise management of patients with SVD. For example, SVD with plaque rupture or erosion may reacted differently to DES or DCB in clinical outcome. Although an observation study demonstrated that DCB is safe and effective for ACS complicated with vulnerable plaque [26]. There is still unknown whether DCB is useful in ACS patients with plaque rupture in SVD. The choice of DES or DCB treating SVD should be based on clinical risk factors, functional assessment and plaque characteristics. Namely, for those patients both with plaque rupture and SVD, whether DCB or DES was more effective remained still unknown.

Small coronary lumen size or area presented with regional hemodynamic change which resulted in distinct atherosclerosis progression compared with the large lumen size [27]. Coronary artery flow velocity was reported to inversely relate to the lumen size and small lumen size may suffer from higher blood flow velocity [28]. Moreover, the previous study revealed that the size of the cavity inside the ruptured plaque was positively related to vessel size [29]. The prevalence of stent restenosis was also higher in small lumen artery than large lumen size [6]. Distinct size of vessel lumen exhibited various reaction to different DES. Previous study revealed sirolimus-Eluting stent acted better than paclitaxel-Eluting Stents by reducing MACE and target lesion revascularization in SVD but not in large vessel disease [30]. Furthermore, the previous study demonstrated that small vessel size was significantly associated with poorer prognosis in patients with STEMI [3]. However, as the development of advanced therapeutic strategy and novel technique of drug-eluting stent and DCB, SVD got similar outcome compared with patients of large coronary vessels [31]. Our results also suggested that vessel size of culprit lesion had less impact on patients’ prognosis. However, the number of patients with SVD in our study is quite small. Thus, whether patients with SVD can benefit from OCT guidance for choosing DES or DCB needs future large sample cohort study.

Numerous studies demonstrated that both plaque phenotype and vessel size were essential factors for treatment strategy in the past few decades [14, 15, 22, 23]. Previous study revealed that patients with plaque rupture showed higher prevalence of no-reflow and severer systemic inflammation which needed intensive antithrombotic and anti-inflammatory treatment [32]. However, patients with plaque erosion might benefit from drug therapy without stent while distal and microcirculation embolism should be noticed [33]. Although OCT enabled to identify plaque phenotypes precisely, it was not widely used in clinics because of its high price and operational complexity. In the current study, the association of culprit vessel diameters and plaque features was revealed and size of culprit vessel might assist to evaluate plaque phenotypes which guided us to product intervention strategy. For example, small vessel with diameters ≤3 mm tended to be plaque erosion which was suitable for drug coated balloon dilation. Culprit lesion with large diameters were prone to presenting plaque rupture and more vulnerable features which needed intensive antiplatelet, cholesterol-lowering an even anti-inflammation therapy.

## 5. Conclusions

The present study suggested that vessel size of culprit lesion is significantly associated with plaque phenotype in patients with STEMI. However, patients with different diameters and plaque phenotypes showed no significant difference of clinical outcomes.

## 6. Limitation

First, this study was a single-centre study with small sample size, more than one fourth of the patients were excluded so that selection bias cannot be excluded. Second, due to adhesion of thrombus in culprit lesion, error may exist in diameter measurement in some cases. Third, some interventional procedures, such as guidewire entry and thrombus aspiration before OCT examination, may change the structure of the underlying plaque. Therefore, some cases of plaque phenotype were misjudged.

## Data Availability

The datasets used and/or analyzed during the current study are available from the corresponding author on reasonable request.
